# Rheumatic heart valve disease: navigating the challenges of an overlooked autoimmune disorder

**DOI:** 10.3389/fcvm.2025.1537104

**Published:** 2025-03-13

**Authors:** Adrien Lupieri, Prabhash K. Jha, Victor Nizet, Walderez O. Dutra, Maria Carmo P. Nunes, Robert A. Levine, Elena Aikawa

**Affiliations:** ^1^ The Center for Excellence in Vascular Biology, Brigham and Women’s Hospital, Harvard Medical School, Boston, MA, United States; ^2^ Department of Pediatrics and Skaggs School of Pharmacy and Pharmaceutical Sciences, University of California San Diego, La Jolla, CA, United States; ^3^ Departamento de Morfologia, Instituto de Ciências Biológicas, Federal University of Minas Gerais, Belo Horizonte, Brazil; ^4^ School of Medicine, Hospital das Clínicas, Federal University of Minas Gerais, Belo Horizonte, Brazil; ^5^ Cardiac Ultrasound Laboratory, Massachusetts General Hospital, Harvard Medical School, Boston, MA, United States; ^6^ Center for Interdisciplinary Cardiovascular Sciences, Division of Cardiovascular Medicine, Department of Medicine, Brigham and Women’s Hospital, Harvard Medical School, Boston, MA, United States

**Keywords:** acute rheumatic fever, rheumatic heart disease, autoimmune disease, neoangiogenesis, lymphangiogenesis

## Abstract

Despite being a leading cause of morbidity and mortality among young people, affecting predominantly women, rheumatic heart disease (RHD) remains neglected and understudied. This autoimmune condition arises from a complex continuum that begins with repeated Group A Streptococcal (GAS) pharyngitis, leading to acute rheumatic fever (ARF) that eventually results in damage to the heart, mainly affecting the mitral valve. While RHD has been nearly eradicated in high-income countries, it continues to be a significant and active health issue in low- and middle-income countries. The resolution of this disease faces several challenges, including the difficulty of diagnosis and the lack of access to preventive measures in resource-poor communities. Addressing these issues will require a global health collaboration involving healthcare professionals, policymakers, and advocacy groups. However, over the past two decades, there has been a revival of scientific interest, fostering optimism for the future. Recent research has significantly advanced our understanding of RHD, shedding light on the immune-to-autoimmune transition, neoangiogenesis, and lymphangiogenesis processes. Additionally, perspectives of discoveries in biomarkers and the development of genetic, transcriptomic, and provide a solid foundation for further advancements in the field.

## 1 Global landscape of rheumatic heart disease

### 1.1 Global impact of rheumatic heart valve disease

ARF is a complication of untreated nasopharyngeal infection by beta-hemolytic GAS, primarily affecting children and adolescents aged 5–15 years. Although ARF symptoms resolve over weeks to months, ARF can progress to rheumatic heart valve disease (RHVD), which remains a leading cause of acquired heart failure and cardiovascular death, impacting around 40 million people and causes roughly 300,000 annual deaths worldwide (1, 2). RHVD is typically viewed as a condition associated with poverty, overcrowding, inadequate sanitation, and limited access to healthcare. Historically, before the socioeconomic developments of the late 20th century, ARF and RHVD were more prevalent worldwide, affecting young people in both high- and low-income nations. Currently, most patients affected by these conditions live in low- and middle-income countries in Central and South Asia, the South Pacific, and sub-Saharan Africa (3, 4). Although RHVD incidence has declined in high-income countries over the past decades, a recent resurgence has been observed in many of these regions. This increase may partly stem from the influx of migrants and refugees from areas with high RHVD prevalence (5). However, a 2008–2018 study on ARF and RHVD in the USA found that most cases were not linked to foreign exposure but rather to poor living conditions and limited healthcare access in underserved communities (6). RHVD also remains prevalent in some high-income countries, including Australia, New Zealand, and Canada, disproportionately affecting indigenous young people. Higher incidence in these populations has been associated with inadequate housing, restricted access to preventive and primary care and challenges in early diagnosis (7). Ongoing migration crises and the marginalization of vulnerable populations worldwide continue to shape RHVD epidemiology and management, reinforcing its status as a pressing global health issue (8).

Furthermore, while ARF appears to be equally found in males and females, RHVD has a two-fold higher prevalence in women than men (9). The cause behind this sex disparity remains unclear and warrants further investigation of underlying factors. In addition to the higher female prevalence, studies indicate that RHVD in pregnancy is a significant non-obstetric cause of maternal mortality, emphasizing the urgent need for enhanced monitoring and management of affected women (10, 11).

In summary, both the socioeconomic environment and genetic susceptibility play crucial roles in the development of the disease. To effectively control it, we must address key challenges, including ensuring access to existing prevention and treatment options, as well as increasing our understanding of the underlying biological mechanisms.

### 1.2 Challenges in the care of ARF and RHVD

Most RHVD-associated morbidity and mortality can be effectively managed through existing therapies. However, the high prevalence of this condition in certain regions is primarily due to limited access to healthcare infrastructure and a lack of public education regarding disease prevention. Addressing these barriers is crucial for reducing the impact of RHD and improving health outcomes in impacted communities, which requires enhancing the treatment of GAS infections, ensuring early detection of subclinical RHVD, providing access to surgical interventions in resource-poor regions, developing biomarkers for early disease, and increasing scientific research.

The most effective strategy for preventing RHVD is the timely and adequate treatment of primary GAS-induced pharyngitis and thereby halting the development of ARF. This approach relies on simple and cost-effective antibiotic treatment, such as penicillin, to prevent recurrent episodes of pharyngitis and reduce the risk of ARF. Even in asymptomatic patients with echocardiogram-detected RHVD, secondary penicillin prophylaxis is effective in preventing disease progression (12).

However, ARF is often underdiagnosed for various reasons, including its diverse clinical manifestations, the symptom overlaps with other common diseases like malaria and viral infections, and a lack of health-seeking behavior. Consequently, many patients are diagnosed too late with RHVD at advanced and severe stages resulting in high mortality rates at a young age (13). It is crucial to improve access to diagnosis and guiding antibiotic prophylaxis in remote and underserved communities, particularly during the period between the initial injury and the onset of clinical disease (10, 14).

The overall prevalence of subclinical RHVD, often called “latent” RHVD, is estimated to be seven to eight times higher than clinically manifested RHVD (15), highlighting the critical importance of early detection in the initial stages of RHVD. Notably, echocardiography has been shown to be four times more sensitive in detecting RHVD compared to traditional cardiac auscultation (15). Therefore, improving access to diagnostic tools is essential for effectively identifying early cases of RHVD. This can be achieved through the implementation of echocardiography-based screening in remote community settings, which will facilitate early detection of valvopathies and enhance patient care.

Mitral regurgitation (MR) is the most prevalent valvular dysfunction during the early stages of RHVD, typically associated with structural impairment of the MV apparatus during acute rheumatic inflammation in children (16). Later disease stages are associated with morphological changes of the MV such as thickening and stiffening of leaflets and chordae, ultimately leading to mitral stenosis (MS) (17). These severe valve dysfunctions are strongly associated with functional limitation and patient mortality, so that surgical or catheter-based interventions are required for repair or replacement of the damaged heart valves. However, in resource-poor settings, health facilities and trained personnel capable of performing percutaneous and surgical interventions may be limited. In some cases, patients diagnosed late with advanced-stage RHVD may receive limited benefits from valve intervention due to a poor prognosis with persistent pulmonary hypertension and right-sided heart failure (9, 18).

### 1.3 Re-emergence of interest

Despite its ongoing prevalence in certain regions, research and funding for RHD have lagged significantly, with clear evidence of disproportionate ratio of research funding relative to the disease burden (19).

**Figure 1:**
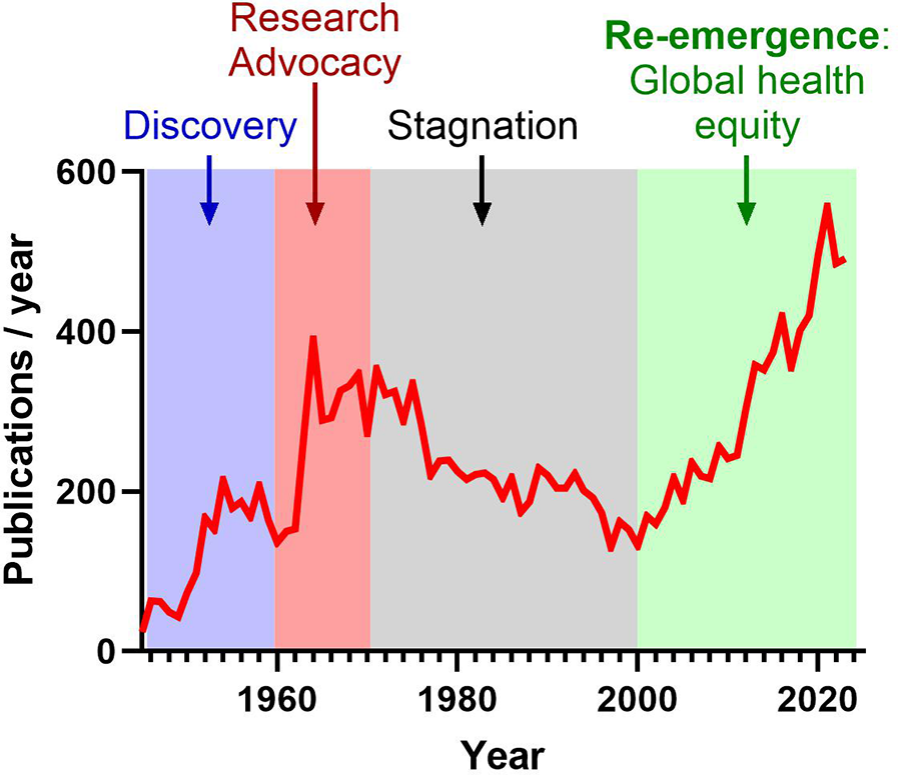
Evolution of publications related to “rheumatic heart disease” referenced in PubMed over the past 80 years. Following an initial phase of significant discoveries, the 1960s witnessed increased advocacy and awareness efforts within the scientific community. However, research stagnated from the 1970s to the 2000s due to the eradication of RHD in high-income countries. Recently, there has been a resurgence of global awareness, underscored by various initiatives and resolutions prioritizing ARF and RHD.

Historically, the first period from the 1930s and 1950s marked significant advances, including the establishment of the first Jones criteria for ARF, some major advances in bacteriology and the emergence of antibiotics to treat GAS pharyngitis along with secondary antibiotic prophylaxis to prevent ARF recurrence. In the 1960s, targeted advocacy, education, and awareness efforts led by the American Heart Association largely promoted global research effort (Figure 1). However, the subsequent reduction of RHVD incidence and severity in high-income countries resulted in prolonged stagnation of research from the 1970s and 2000s (Figure 1) (20). Recent years have seen the re-emergence of global awareness regarding RHVD (Figure 1), emphasized by several initiatives in Africa, including Drakensberg Declaration (2005) (21), Mosi-o-Tunya Call to Action (2014) (22), Cairo Accord on RHD (2017) (23), Cape Town Declaration on Access to Cardiac Surgery in the Developing World (2018) (24).

The surge in research interest related to RHD after 2000 can be largely attributed to increased global health prioritization and a focus on health equity (Figure 1). International organizations like the World Health Organization (WHO) and World Heart Federation (WHF) recognized RHD as a major cause of cardiovascular morbidity and mortality, particularly in low- and middle-income countries. This led to heightened awareness, more research, and greater funding for prevention and treatment initiatives. At the same time, attention shifted toward the social determinants of health, with a focus on how poverty, limited healthcare access, and inequities disproportionately affect RHD outcomes, driving further research on its persistence in underserved populations (25, 26). Additionally, In May 2018, the World Health Assembly adopted a resolution (WHA71.14) prioritizing the global response to ARF and RHVD. In 2020, the American Heart Association has issued a call to action aimed at reducing the global impact of RHVD (27). Most recently, in September 2023, the National Institutes of Health, through the National Institute of Allergy and Infectious Diseases and the National Heart, Lung, and Blood Institute, released a notice of special interest (NOT-HL-23-106) aimed at advancing RHVD research. Furthermore, the Leducq Foundation, an international charitable organization, is dedicated to supporting collaborative research on RHVD. This organization provides funding for international networks with three primary goals: identifying biomarkers for ARF, developing a vaccine against GAS, and creating a polymeric heart valve for patients suffering from advanced RHVD.

Collectively, these global initiatives and funding sources are expected to drive significant advancements in research on ARF and RHVD, emphasizing innovative approaches and the development of effective strategies to prevent and cure RHVD.

## 2 From immunity to autoimmunity: mechanisms underlying ARF and RHVD

**Figure 2:**
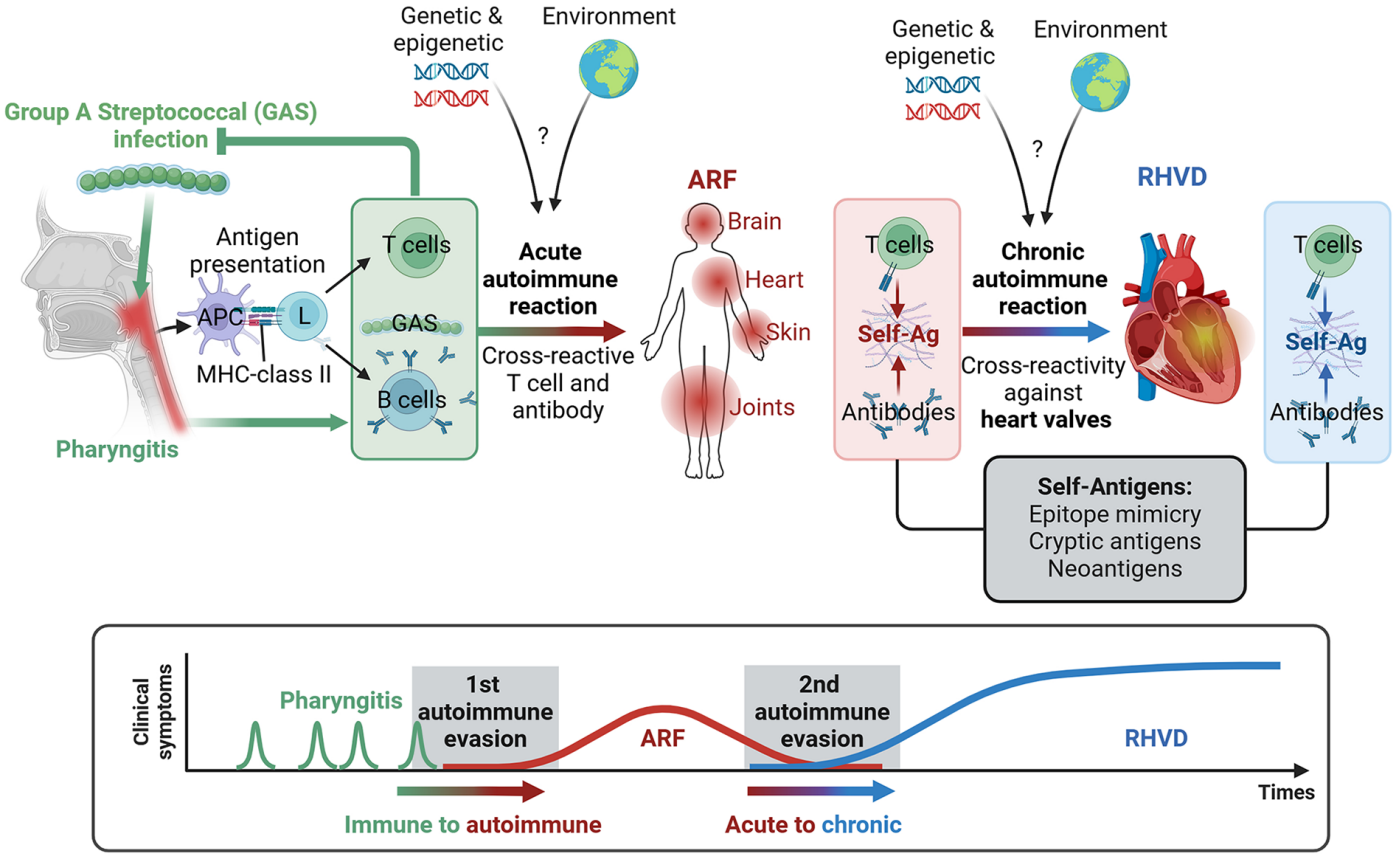
Continuum of disease from group A streptococcus (GAS) infection to autoimmune acute rheumatic fever (ARF) and rheumatic heart valve disease (RHVD). Following GAS pharyngitis, the adaptive immune system processes bacterial antigens, and antigen-presenting cells (APCs) activate lymphocyte responses. T cell clones that recognize GAS epitopes are activated alongside B cells, which produce antibodies targeting GAS. Repeated episodes of GAS pharyngitis, combined with genetic and environmental risk factors, can trigger an autoimmune response affecting the brain, heart, skin, and joints through the cross-reactivity of self-antigens (self-Ag). This autoimmune response may lead to long-term damage, specifically to the cardiac valves, resulting in RHVD. The transition from acute to chronic autoimmune reactions, particularly those targeting the heart valves, may be linked to the evolution of self-antigens, including the release of cryptic antigens and the formation of neoantigens.

A single GAS infection triggers a natural immune response. However, repeated or untreated infections can occasionally shift this to an autoimmune reaction, leading to ARF, which affects various organs, including the joints, brain, skin, and heart (Figure 2). This autoimmune response can cause long-term damage to the cardiac valve, known as RHVD. It is established that in this condition triggers an immune response that alters the matrix architecture and cellular components of tissues, particularly the MV leaflets (28, 29). This alteration of MV function is observed in up to 60% of ARF cases, leading to disruption of the normal blood flow through the heart chambers, which can result in heart failure and pulmonary hypertension.

### 2.1 Acute rheumatic fever, group A Streptococcus and molecular mimicry

ARF is an acute autoimmune response triggered by the infection with group A Streptococcus (GAS). It is estimated that over 600 million cases of symptomatic GAS pharyngitis occur annually (25). However, only 3% to 6% of patients infected by GAS develop ARF (4), indicating that factors such as genetic susceptibility, socioeconomic conditions, and the virulence of GAS may play a role in its development. Patients with ARF can experience a variety of clinical manifestations that may affect the skin, subcutaneous tissues, joints, heart, and brain, though these effects can vary significantly among individuals.

**Figure 3:**
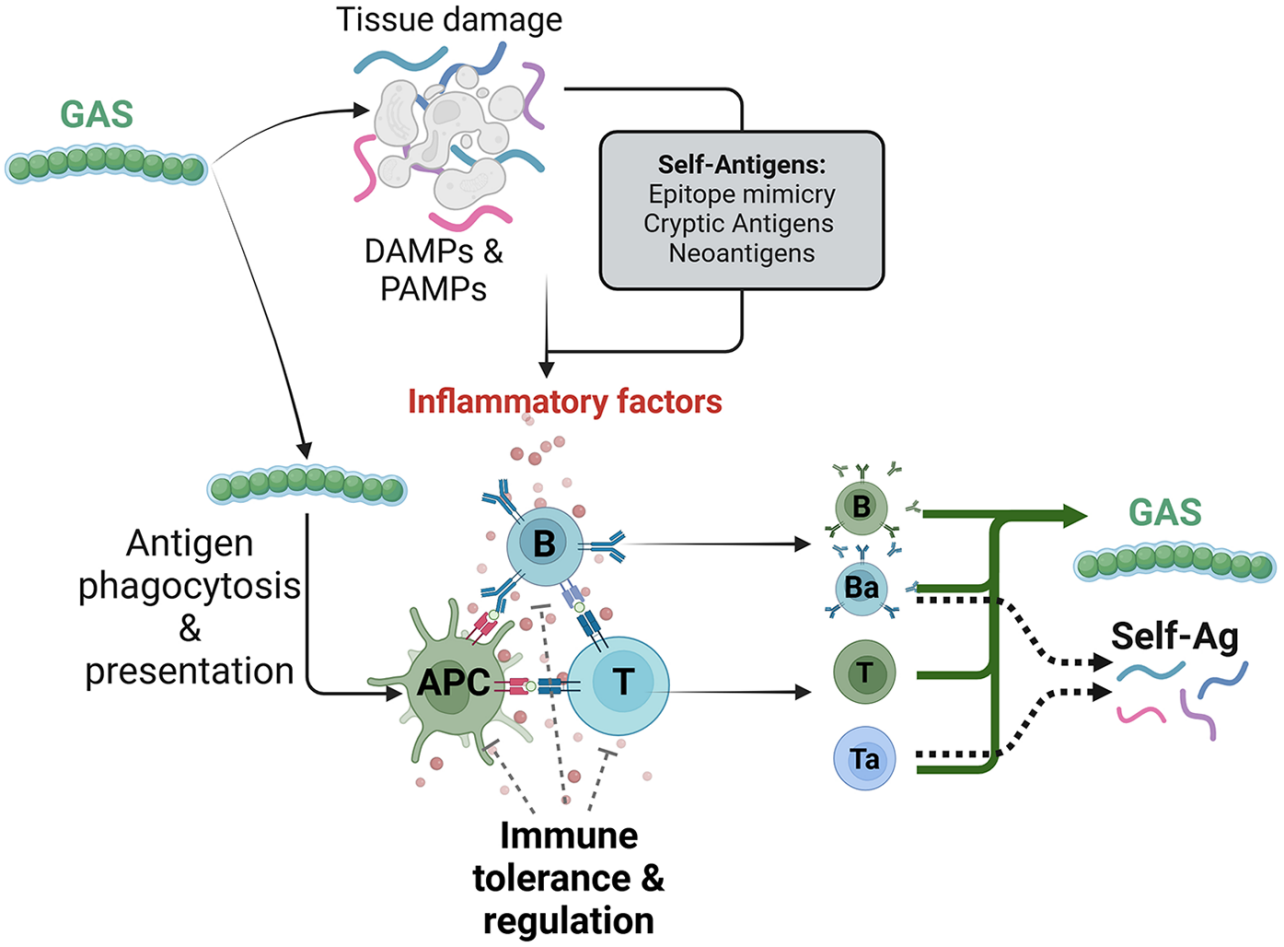
Generation of autoimmune immune response by group A streptococcus (GAS) infection. GAS infection triggers an immune response that leads to tissue damage and the release of damage- and pathogen-associated molecular patterns (DAMPs and PAMPs). Concomitantly, GAS antigens are processed and presented by antigen presenting cells (APCs), activating reactive T and B cell clones. However, the combination of repeated infectious episode and a deficiency in immune tolerance and T cell regulation processes, along with the generation of cross-reactive self-antigens, may contribute to the maturation and activation of autoreactive clones of T cells (Ta) and B cells (Ba), which react to both GAS and self-antigens.

Primary GAS pharyngeal infection induces innate immune reaction and phagocytosis of GAS by antigen-presenting cells, such as macrophages and dendritic cells, for further antigens presentation, and subsequent clone selection and activation of both T and B cells (17). Consequently, the initiation of the adaptive immunity through the activation of specific repertoire of activated T cells and B cells will fight the primary infection targeting GAS-specific antigens (Figures 2, 3). However, the mechanism turning the immune reaction targeting GAS into an autoimmune reaction affecting host tissues is not entirely understood.

Among the key mechanisms driving autoimmunity, molecular mimicry is the most frequently reported (8, 30–32). The current paradigm of ARF suggests that the similarity of epitopes allows T cells and B cells to recognize both GAS-specific antigens and tissue specific self-proteins through receptors (T-Cell Receptors, TCR; B-Cell Receptors, BCR). This cross-reactivity leads to the production of antibodies that target not only GAS-specific antigens, but also self-proteins found in the brain, heart, skin, and joints.

Several examples of molecular mimicry have been identified in the various organs affected during ARF. For instance, neurological manifestation (chorea syndrome) is associated with cross-reacting antibodies for group A carbohydrate epitope N-acetyl-β-d-glucosamine (GlcNAc) and antigens found in the brain such as the D1 and D2 dopamine receptors, lysoganglioside and tubulin (17). Rheumatic carditis has been associated with cross-reactive antibodies targeting group A carbohydrate or streptococcal M protein and cardiac myosin and valvular laminin (17). In addition, collagen-reactive antibodies and T cells have also been observed in patients with ARF, potentially due to epitope similarities with M protein (33) or streptococcal collagen-like proteins (34). Other studies suggested that these anti-collagen antibodies are induced by the exposure of cryptic collagen epitopes resulting from collagen degradation or the aggregation of collagen by certain streptococcal serotypes (17).

Nevertheless, epitopes mimicry is commonly found in nature, but cross-reactivity requires self-reactive lymphocytes to escape the robust mechanism of thymic and peripheral tolerance (35). This process of immune tolerance prevents lymphocytes with a high affinity for self-antigens from functional differentiation through several mechanisms. These include inhibitory molecules that downregulate immune responses (such as cytotoxic T-lymphocyte associated protein 4, CTLA-4; Programmed cell Death protein 1, PD-1); anergy, where T cells become unresponsive without co-stimulatory signals; ignorance, which keeps certain self-antigens hidden from the immune system; and active suppression by regulatory T cells (Tregs) that inhibit autoreactive lymphocytes (Figure 3). Together, these mechanisms help maintain self-tolerance and protect against autoimmune diseases (36). However, lymphocytes with low affinity for self-antigens that react weakly, can escape thymic selection and persist in the periphery (37), potentially becoming activated through repeated stimulation and loss of tolerance. In this model, in which a clone of lymphocyte reacting to GAS infection but also to self-antigen(s) with low affinity are activated and able to mature into memory lymphocyte, recurrent GAS pharyngitis episodes might increase its reactivity and lead to ARF through the recognition of self-antigens (Figure 3). Mathematical modeling of autoimmune disease initiated by pathogen infection has suggested that cross-reactivity of antigen should be minimal to escape the processes of negative selection and T-cell activation, but needs to be strong enough for eliminating pathogenic antigens (38).

Moreover, some hypotheses suggest that specific strains of GAS may play a role in the induction of ARF. The virulence of these GAS strains has been linked to the high variability of the M protein present on their surface (39). This variability may induce different potential cross-reactivity with self-antigens. However, a definitive identification of rheumatogenic GAS strains has not been established. Additionally, other theories proposing that neo-antigens are formed from the combination of the PARF motif on the M protein bound to the CB3 region of type IV collagen (40). The immunogenicity of this chimeric complex leads to the production of autoantibodies targeting type IV collagen, possibly cross-reacting with other collagen types, including type I collagen present in the valve.

### 2.2 RHVD: transition from acute to chronic autoimmunity

Rheumatic carditis affects both the myocardium and the heart valves. In the early stages of the disease, pan-carditis is observed. However, as the disease progresses, it predominantly impacts the left heart valves, particularly the MV, leading to RHVD. However, the underlying pathological mechanisms of this preferential MV involvement during the chronic phase remain unclear.

**Figure 4:**
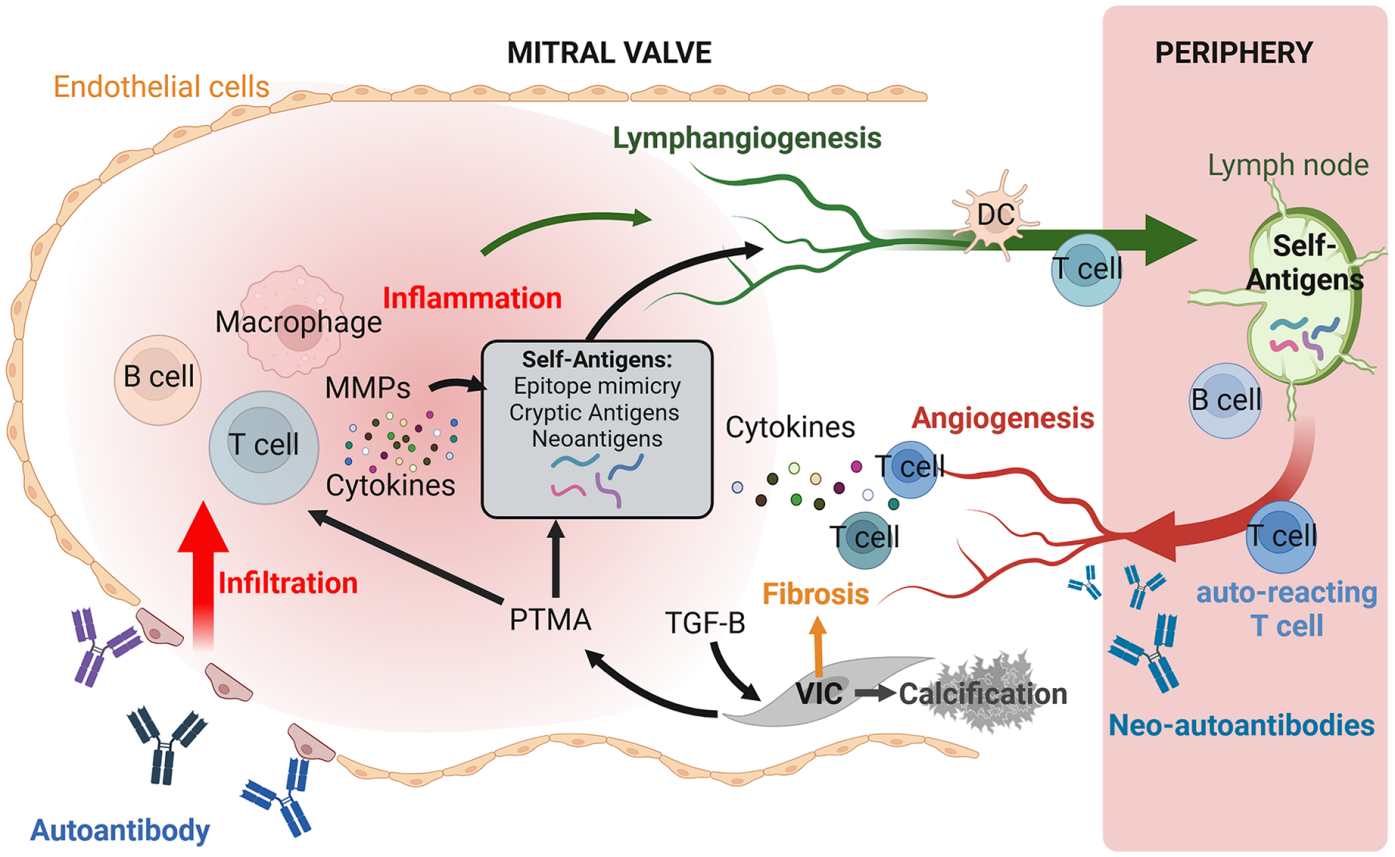
Schematic representation of the pathogenesis of rheumatic heart valve disease (RHVD). Antibodies targeting Group A Streptococcus (GAS) epitope can cross-react with autoantigens of the valve endothelium by molecular mimicry, leading to endothelial activation and subsequent infiltration of immune cells, including macrophages, B cells, and T cells. The local inflammation, coupled with the presence of autoantibodies cross-reacting with valve components, promotes the release of cytokines and matrix metalloproteinases (MMPs), contributing to the generation of new self-antigens (both cryptic and neoantigens). Additionally, inflammatory factors such as TGFβ, promote valvular interstitial cell (VIC)-mediated fibrosis and calcification, leading to valve dysfunction. The formation of neovessels within the inflamed leaflets may play a crucial role in sustaining chronic inflammation in the mitral valve (MV) by supporting the trafficking of immune cells, antigen-presenting cells (APCs) and self-antigens through lymphatic neovessels to the lymph nodes. This process allows autoreactive cells to recirculation into the bloodstream and subsequently remigration to the leaflets via neocapillaries. Furthermore, local factors, such as prothymosin alpha (PTMA), may enhance autoimmune responses by increasing T cell activity and their ability to bind collagen.

Rheumatic MV damage is initiated through the binding of GAS-specific antibodies that cross-react with valvular endothelium molecules, including laminin and other glycoproteins. This interaction activates valvular endothelial cells, leading to the upregulation of vascular cell adhesion molecule 1 (VCAM-1) on the cell surface. The subsequent rearrangements in the endothelium facilitate immune cell infiltration (Figure 4) (17, 41). The resulting intra-valvular inflammation, supported by chronic activation by self-antigens, produces cytokines, cytotoxins and proteases that contribute to structural alterations in the valve tissue, ultimately causing valve dysfunction.

Similarly to the triggering of the acute autoimmune reaction, the chronic phase has been associated with immune cross-reactivity. While only cardiac myosin and M protein mimicry have been associated with cardiac valvulitis in animal studies (42, 43), other forms of molecular mimicry likely play a significant role in triggering rheumatic carditis. This include the exposure of cryptic collagen epitopes (17), and the molecular mimicry between type I collagen-derived peptides and GAS epitopes (33). However, another study reported the presence of autoantibodies targeting collagen I in patients with ARF, although these antibodies do not cross-react with GAS-derived antigens (44). This lack of cross-reactivity suggests an alternative mechanism for autoimmunity that operates independently of molecular mimicry (Figure 4).

Recent studies have revealed that protein prothymosin-alpha (PTMA) levels are elevated in the mitral valves of RHVD patients. This protein has been shown to promote both CD8+ T-cell cytotoxicity and recognition of human type I collagen via Very Late Antigen α2β1 (VLA-2), suggesting a potential role in promoting autoimmune responses (33).

### 2.3 Angiogenesis and lymphangiogenesis

Cardiac valve leaflets are largely avascular structures under normal physiological conditions as thin leaflets get their nutrients through the process of diffusion. Neovascularization has been described as a key feature of valvular heart disease, including RHVD (41, 45–47). This phenomenon could provide valuable mechanistic insights into the progression of RHVD. The primary functions of neovessels are to supply oxygen, metabolites, and support immune cell trafficking. This process is regulated by several growth factors including vascular endothelial growth factors (VEGFs), and by extracellular matrix remodeling, which aids endothelial cell migration.

However, dysfunctional angiogenesis can also compromise valve tissue structure and mechanical properties and could lead to blood leakage into the tissue. Of note, immature vessels were observed in human RHVD MVs (41), suggesting that aberrant vessel formation may contribute to disease pathogenesis, either by disrupting the normal valve architecture or supporting infiltration of immune cells and soluble inflammatory factors. Furthermore, studies have demonstrated that preventing angiogenesis may serve as a potential therapeutic strategy to mitigate valve disease (46), emphasizing the crucial role of valvular neoangiogenesis in the progression of RHVD (Figure 4).

The role of lymphatic vessels in the progression of valvular disease is not yet well established. The lymphatic system consists of blind-ended vessels that form a one-way network essential for draining interstitial fluid and supporting adaptive immune responses (48). Its primary immune function is to transport antigens and activated antigen-presenting cells (APCs) to the lymph nodes, thereby supporting the activation of adaptive immune effector cells and humoral responses (Figure 4). Remarkably, de-novo lymphangiogenesis has been implicated in chronic inflammation, as seen in conditions such as psoriasis, rheumatoid arthritis, and allograft transplantation (49). These conditions often feature significant lymphangiogenesis and immune cell trafficking mediated by vascular endothelial growth factor receptor 3 (VEGFR3)-dependent CCL21 production (50). In such cases, inhibiting VEGFR-3 has been shown to reduce lymphangiogenesis and decrease CCL21-mediated adaptive immune responses (49, 50).

Moreover, while the development of lymphatic vessels in mitral valves of patients with RHVD has been previously observed (41, 51, 52), a recent study has elegantly characterized the development of lymphatic vessels (LYVE1+) in the mitral valves during autoimmune valvular carditis in K/B.g7 mice (53). These mice naturally produce autoantibodies targeting the widely expressed glycolytic enzyme glucose-6-phosphate isomerase (GPI), which leads to severe autoimmune arthritis and valvular carditis similarly to human ARF and RHVD (54).

## 3 Conclusion and future considerations

### 3.1 Sexual discrepancy in ARF and RHVD

Although ARF appears to affect males and females similarly (17), RHVD is more prevalent in women (relative risk of 1.6–2) (9). Epidemiological data on RHVD align with patterns observed in other autoimmune diseases, which have an approximately two-fold higher prevalence in women (36). This sexual difference has been linked to sex hormones and the X chromosome. Estrogen, the predominant female sex hormone, is known to promote antibody and autoantibody responses in females through B cell activation, hypermutation, and class switch recombination (55). Importantly, estrogen has been found to regulate T cell development and enhance the immune response by increasing the expression of pro-inflammatory factors (55). Additionally, it decreases the expression of the autoimmune regulator (AIRE) gene, thereby increasing the susceptibility to developing autoreactive lymphocyte (55). This dysregulation can contribute to the development of autoimmune disorders (36).

The presence or absence of a second X chromosome is strongly associated with susceptibility to autoimmune disease, as the inconsistency of the X chromosome inactivation induces an imbalance of gene expression, affecting approximately 15% of X-linked genes (56). Several of these escaped X-linked genes are involved in various processes of immune activation and regulation (e.g., CD40l, CXCR3, OGT, FOXP3, TLR7, TLR8, IL2RG, BTK, and IL9R) (57). Similarly, other regulatory mechanisms, such as non-coding transcripts, could play important roles. For instance, XIST lncRNA, a key regulator of the X-chromosome inactivation, has been shown to promote the splenic expression of TLR7, TLR8 and CXCR3, and increase levels of TNF, IL-1β, and IL-6 (58). Furthermore, it is estimated that the X-chromosome carries approximately 10% of non-coding miRNA, and several estrogen-regulated miRNA has been identified, which may be an important source of sex discrepancies and warrants further investigation (55).

The importance of these sex-dependent mechanisms in RHVD remains unexplored. However, new insights into the sex-specific pathobiology of RHVD are gradually emerging. Recent findings highlight that the stimulation CD8+ T-cell cytotoxicity by PTMA was linked with estrogen receptor-alpha activity, thus suggesting a plausible role of this and other factors in the sex predisposition in RHVD (33).

### 3.2 Genetic susceptibility of ARF and RHVD

In addition to socioeconomic and environmental risk factors, current knowledge indicates a strong genetic susceptibility to ARF. A study on twin pairs estimated the heritability component of susceptibility to ARF at 60% (59). Epidemiologic studies reported that among the more than 600 million cases of symptomatic GAS infection, only 3%–6% of individuals will develop ARF (60). This suggests that the autoimmune disorder only develops in genetically predisposed individuals. Furthermore, it is estimated that 60% of patients with ARF will develop RHVD. Although there is a well-established association between GAS infection and RHVD, RHVD seems to be induced autonomously even after the initial stimulus is removed, thus suggesting that host-factors, such as genetic and epigenetic influences, play a crucial role (25). However, risk factors promoting the transition from acute to chronic autoimmune condition remain unknown. Therefore, genetic studies to identify individuals at high-risk genes could help in developing effective prevention and control strategies, enhancing the management of RHVD.

Genetic susceptibility for RHVD development has been studied in genome-wide association studies (GWASs). Significant association have been demonstrated in class II human leukocyte antigen (HLA) loci (HLA-DQA1, HLA-DQB1, HLA-DRB1) (60), which are expressed on the surface of APCs and support T cell receptor-mediated immune responses. Additionally, class I HLA (HLA-B) has been associated with ARF (60). Similarly, immunoglobin heavy locus (IGHV4-61) was linked to an increased risk of RHVD in Oceanian countries (60).

Furthermore, several polymorphisms in genes coding for immune-related proteins have been associated with ARF and RHD susceptibility. These proteins include toll-like receptor 2 (TLR-2), interleukin 1 beta (IL-1β), interleukin 6 (IL-6), interleukin-10 (IL-10), tumor necrosis factor alpha (TNF-α), transforming growth factor beta 1 (TGF-β1), angiotensin I converting enzyme (ACE), mannose-binding lectin 2 (MBL2), and mannose-binding protein-associated serine protease 2 (MASP2) (61, 62). Although other gene variants have been associated with ARF or RHVD, most have been observed only in single studies, making further meta-analysis impossible (61).

Published GWASs in ARF/RHVD are relatively small and lack the statistical power to characterize reliable and reproducible risk factors, underscoring the need for large-scale, multicenter studies across diverse populations. Additionally, current research has disproportionately focused on European and Asian cohorts, with limited assessment of sub-Saharan populations, who are dramatically affected by these diseases. Among the promising upcoming studies, the RHDGen network GWAS study stands out. It combines echocardiography screening for enrolling RHVD cases and ethnically matched controls, a genetic trio (both parents and offspring) replication, and plasma collection. This presents a unique opportunity to identify relevant genetic factors and biomarkers associated with differential RHD risk in African populations (63, 64). The emergence of robust new knowledge on the genetic susceptibility to RHVD will pave the way for more precise, effective, and individualized prevention and treatment strategies.

### 3.3 Key strategies/future directions to combat ARF and RHVD

The major strategies the scientific community can implement to combat the ARF and RHD can be divided into five key areas: (I) Development of diagnostic tools and strategies, including accessible echocardiography to identify prognostic tissue properties (65), point-of-care rapid tests and portable methods for detecting GAS-antigens, and biomarkers for identifying ARF and latent RHVD. The emerging artificial intelligence-based classification of echocardiography could also provide robust support for detecting early ARF and latent RHVD (66), (II) Large scale epidemiologic and genetic studies of ARF and RHVD with aims to characterize demographic features, outcomes, and genetic markers. (III) Multi-omics approaches, integrating proteomics and transcriptomics at both tissue and single-cell levels (e.g., plasma, peripheral blood mononuclear cells, and valve tissue) are essential to provide functional understanding of the disease. The emergence of open-source omics datasets will provide opportunities for secondary and meta-analysis, even in resource-poor settings. Moreover, single cell omics will help appreciate these changes in the cellular landscape of the disease. Multimodal analysis will offer crucial insights into potential biomarker candidates and pathophysiology, paving the way for new therapeutic strategies, including CRISPR-based gene editing therapies targeting immune cells (67). (IV) Development of preventive treatment, including long-acting penicillin to improve secondary prophylaxis and the development of a vaccine against GAS to transform the primary prevention. (V) Development of education and awareness campaigns to improve affected population understanding of the danger of GAS infection, ARF and RHVD. These initiatives should be implemented through a variety of multimedia campaigns (printed materials, social media, television, radio), school-based educational programs, and training for community workers with hand-held ultrasound screening (68, 69).

These efforts collectively aim to enhance the early detection, prevention, and management of ARF and RHD. Addressing these challenges requires sustained commitment from healthcare professionals, policymakers, and advocacy groups, including non-governmental organizations, to drive long-term change and improve outcomes worldwide.
